# On Free Energy Calculations in Drug Discovery

**DOI:** 10.1021/acs.accounts.5c00465

**Published:** 2025-10-10

**Authors:** Alessia Ghidini, Eleonora Serra, Andrea Cavalli

**Affiliations:** † Centre Européen de Calcul Atomique et Moléculaire (CECAM), 27218 Ecole Polytechnique Fédérale de Lausanne, 1015 Lausanne, Switzerland; ‡ Department of Pharmacy and Biotechnology (FaBiT), Alma Mater Studiorum - University of Bologna, via Belmeloro 6, 40126 Bologna, Italy; ¶ Computational & Chemical Biology, 121451 Fondazione Istituto Italiano di Tecnologia, via Morego 30, 16163 Genoa, Italy

## Abstract

This Account discusses recent progress and challenges in binding free energy computations, focusing on two classes of enhanced sampling techniques: alchemical transformations and path-based methods. Binding free energy is a crucial metric in drug discovery, as it measures the affinity of a ligand for its target receptor. Free energy and affinity guide the ranking and selection of potential drug candidates. The theoretical foundations of free energy calculations were established several decades ago, but their efficient application to drug-target binding remains a grand challenge in computational drug design. The main obstacles stem from sampling issues (as binding is a rare event), force field accuracy limitations, and simulation convergence. Alchemical transformations are now the most used methods for computing binding free energies in the pharmaceutical industry. However, while they efficiently calculate energy differences, the application of these methods is often limited to relative binding free energy calculations. Absolute and accurate (error < 1 kcal/mol) binding free energy predictions remain one of the great challenges for computational chemists and physicists. Another limitation of alchemical methods is that they lack the ability to provide mechanistic or kinetic insights into the binding process, crucial for optimizing lead compounds and designing novel therapies. Path-based methods offer, in principle, the possibility to accurately estimate absolute binding free energy while also providing insights into binding pathways and interactions.

This Account explores recent advances in binding free energy methods for drug-target recognition and binding. In particular, we discuss the similarities and differences between alchemical and path-based approaches, highlighting recent innovations in both families of methods and providing perspectives from our group’s contributions. We examine the foundational role of alchemical methods, which have been employed since the inception of free energy calculations, in both equilibrium and nonequilibrium contexts. We also emphasize the growing importance of path-based methods in drug discovery and their ability to predict binding and unbinding pathways, free energy profiles, and binding free energy estimates. In particular, the combination of path methods with machine learning has proven to be a powerful means for accurate path generation and free energy estimations. Building on our recent research, we discuss several path-based applications for drug discovery. Moreover, we focus on two semiautomatic protocols representing our group’s state-of-the-art in free energy calculations. The first protocol is based on MetaDynamics simulation. From this, a recent innovation is instead based on nonequilibrium simulations combined with nonequilibrium estimators. We discuss in depth the advantages and drawbacks of equilibrium and nonequilibrium approaches to drug-target binding free energy predictions.

## Graphical abstract

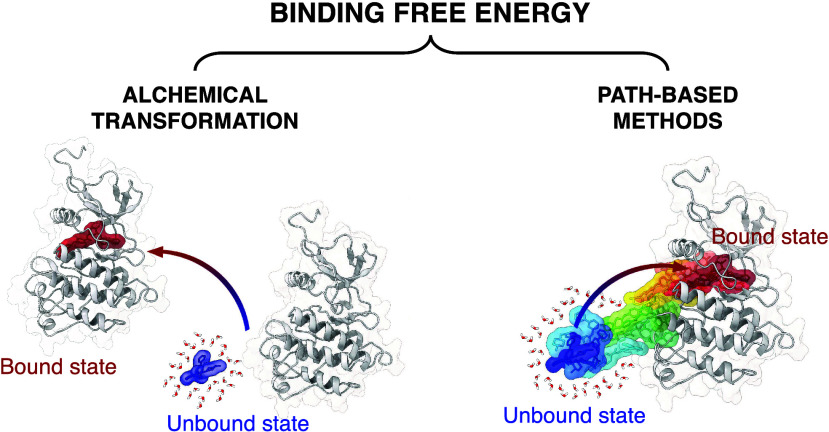

## Key References

-

Bertazzo, M. ; Gobbo, D. ; Decherchi, S. ; Cavalli, A. Machine learning and enhanced sampling simulations for computing the potential of mean force and standard binding free energy. J. Chem. Theory Comput. 2021, 17, 5287–5300 34260233 10.1021/acs.jctc.1c00177PMC8389529.^1^ This work introduces a new semiautomatic computational pipeline for estimating standard binding free energies, based on MetaDynamics and path collective variables.^1^
-

Ghidini, A. ; Serra, E. ; Decherchi, S. ; Cavalli, A. Bidirectional path-based non-equilibrium simulations for binding free energy. Mol. Phys. 2025, 123, e2374465 .^2^ This study presents an updated version of the semiautomatic computational pipeline that integrates path collective variables with bidirectional nonequilibrium simulations. This new protocol allows for straightforward parallelization, significantly reducing the time-to-solution for binding free energy calculations.^2^

## Introduction

Potential drug candidates are ranked based on various parameters, among which is their binding affinity for a specific biological target, typically a protein receptor but increasingly also RNA.

Binding affinity can be determined with several experimental techniques, including biophysical methods such as surface plasmon resonance and isothermal titration calorimetry, as well as biochemical methods like radioactive and enzymatic assays. With the rise in computer power and the advancement of Graphics Processing Units (GPUs), computational methods for estimating binding affinity have gained prominence. In this context, computer-aided drug design holds the promise of reducing both the cost and time associated with the development of new drugs.^3^

The binding affinity of a drug to its target, *K*_*a*_, is directly related to the binding free energy (*ΔG*_*b*_), which is the difference in free energy between the bound state (i.e., the binary ligand-protein complex) and the unbound state (i.e., the free ligand and protein forms). This relationship is expressed by the following equation:

$$\Delta G_{b}^{{}^{\circ}}=-\mathrm{RT}\;\ln\left(K_{a}C^{{}^{\circ}}\right)$$ 1

where *R* is the gas constant, *T* is the temperature and *C*° is the standard-state concentration (1 mol/L). Consequently, free energy calculations have become invaluable tools for estimating *ΔG*_*b*_, and thereby binding affinity, in biochemistry and pharmaceutical research.

The theoretical basis of free energy calculations was established decades ago. The paper by John Kirkwood from 1935 laid the groundwork for two important methods: free energy perturbation (FEP) and thermodynamic integration (TI).^4^ Early free energy calculations were based on analytical theories, as in the case of free energy perturbation formulated by Zwanzig in 1954 relying on perturbation theory.^5^ With advances in computational technology, numerical simulations based on statistical mechanics became feasible.^6^ Today, free energy calculations in drug discovery mainly rely on all-atom Molecular Dynamics (MD) simulations and can be divided into two main categories: (i) alchemical transformations, and (ii) path-based or geometrical methods. A schematic representation of both methods is shown in Figures 1 and 2.

**Figure 1:**
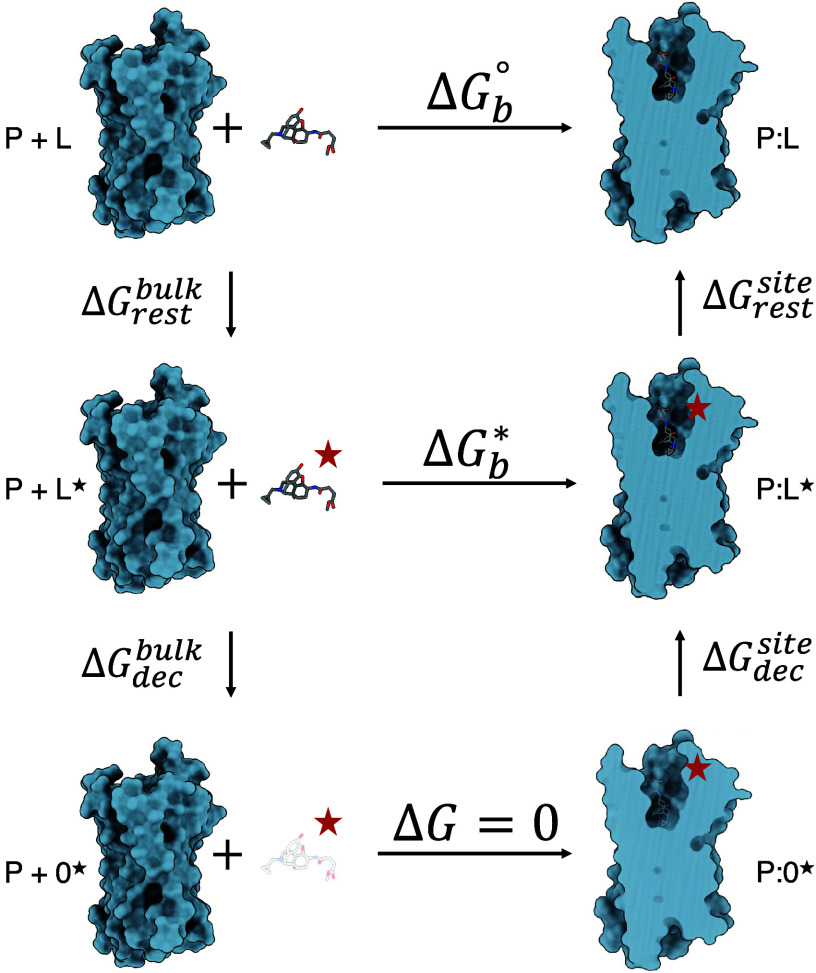
Schematic representation of a thermodynamic cycle using alchemical double decoupling for absolute binding free energy calculations. To prevent the ligand (L) from wandering away from the binding site when decoupled from the protein (P), the ligand is restrained in its bound conformation, position, and orientation (denoted by ★). Then, the restrained ligand is decoupled from both the protein and the bulk solvent (denoted by 0). The free energy contribution due to the addition of the restraints must be evaluated, and following the thermodynamic cycle, the binding free energy is estimated.

**Figure 2:**
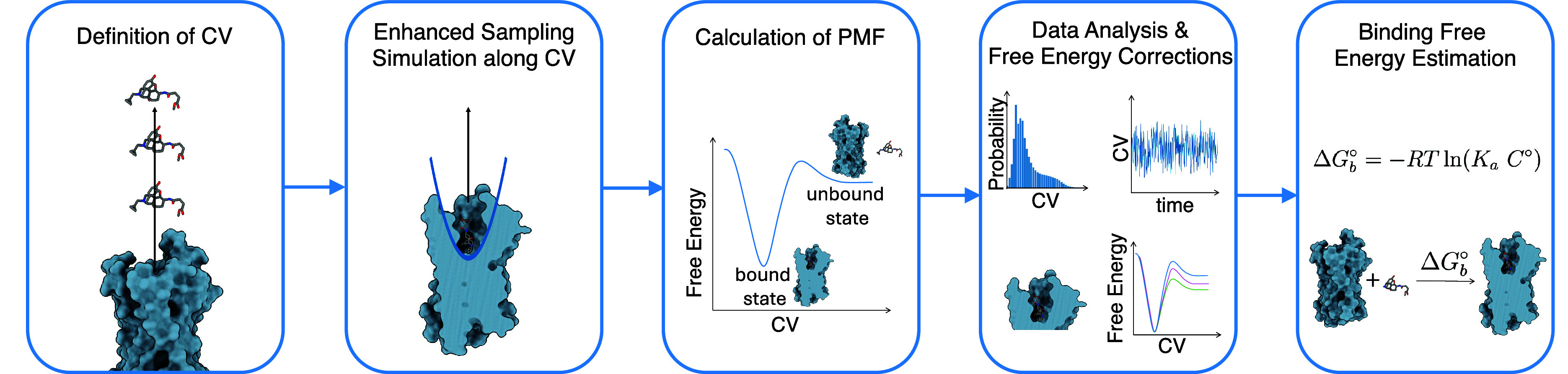
Schematic representation of a workflow for calculating absolute binding free energies using path-based methods. First, a suitable CV is identified. Then, enhanced sampling simulations are performed along the CV. From these simulations, and possibly with free energy estimators, the potential of mean force along the CV is calculated. Subsequent steps include convergence analysis, visual inspection of trajectories, and estimation of protocol-specific free energy corrections. Finally, the binding free energy is estimated.

## Coupling Parameters and Collective Variables

To describe the extent of a chemical reaction, De Donder introduced the concept of degree of advancement of a process in 1920.^7^ Starting from this idea, Kirkwood defined the *order parameter* of a reaction as a variable chosen to describe changes in the system, approximating the reaction coordinate. Alchemical transformations are based on the coupling parameter (λ), an order parameter that describes the interpolation between the Hamiltonians of the initial and final states. Differently, in path-based free energy calculations, order parameters are defined as collective variables (CVs), ξ, whose variations serve as an index of the evolution of the process under investigation. CVs are described by relevant degrees of freedom of the system and can range from simple metrics, such as distances, angles, and RMSD, to more complex observables to capture large-scale motions. The result of path-based calculations is often the potential of mean force (PMF), i.e., the free energy profile along the selected CV.

The design of effective CVs is crucial to accurately capture the system behavior. Ideally, the chosen CV should be representative of the system committor, i.e., the probability that a given configuration will reach the product state before returning to the reactant state. However, the committor is often unknown and high-dimensional, so simpler CVs can approximate it. In particularly challenging transformations such as protein–ligand binding processes, CVs based on simple metrics may fail to represent the correct dynamics of the process. In these cases, more sophisticated CVs may be necessary to describe the relevant degrees of freedom of the system. Notable examples include CVs that represent collective dynamics, such as Path Collective Variables (PCVs),^8^ or those obtained through dimensionality reduction techniques such as Principal Component Analysis (PCA).^9^

### Path Collective Variables

PCVs, introduced by Branduardi et al.,^8^ are two CVs, namely *S*(*x*) and *Z*(*x*), that describe the evolution of a system relative to a predefined pathway in the configurational space. The reference pathway consists of a string of consecutive conformations of the system from an initial to a final state. *S*(*x*) measures the system progression along the high-dimensional pathway, and *Z*(*x*) quantifies deviations orthogonal to the pathway:

$$S\left(x\right)=\frac{\overset{p}{\underset{i=1}{\sum }}ie^{-\lambda ∥x-x_{i}{∥}^{2}}}{\overset{p}{\underset{i=1}{\sum }}e^{-\lambda ∥x-x_{i}{∥}^{2}}}$$ 2

$$Z\left(x\right)=-\lambda^{-1}\;\ln\overset{p}{\underset{i=1}{\sum }}e^{-\lambda ∥x-x_{i}{∥}^{2}}$$ 3

Here, *p* denotes the number of reference configurations included in the predefined pathway, λ is a smoothness parameter, and ∥*x* – *x*_*i*_∥^2^ quantifies the distance between the instantaneous microscopic configuration (*x*) and the *i*th structure in the pathway (*x*_*i*_). Different distance metrics can be used to quantify this deviation, with the mean-square deviation being a common choice. The combination of MD with free energy algorithms and PCVs has proven to be highly effective in studying large-scale conformational transitions and ligand binding to flexible targets in realistic biological systems, as demonstrated in refs 1, 2, and 10. In a recent work, we demonstrated that is possible to rigorously map the protein–ligand binding onto a curvilinear pathway and use PCVs as collective variables to accurately calculate the binding free energy.^11^

## Alchemical Transformations

FEP and TI remain today valuable methods for calculating *ΔG*_*b*_ with all-atom MD simulations involving alchemical transformations. Alchemical transformations sample the process from an initial state (A) to a final state (B) through nonphysical paths. Notably, this does not affect the results, as free energy is a state function and hence is independent of the specific path followed during the transformation.

Alchemical transformations rely on a hybrid Hamiltonian, commonly defined as a linear interpolation of the potential energy of states A and B through a coupling parameter λ:

4 $$V\left(q;\lambda \right)=\left(1-\lambda \right)V_{A}\left(q\right)+\lambda V_{B}\left(q\right)$$

where 0 ≤ λ ≤ 1, with λ = 0 corresponding to state A and λ = 1 to state B.

From a practical point of view, the system is sampled at several different values of λ, using stratification strategies to improve convergence. From this sampling, TI and FEP can be used to determine the free energy difference.

In TI, the derivative of the free energy difference between the initial and final states, *ΔG*_*AB*_, is obtained by integrating along λ the equilibrium average over configurations with a specific λ:

5 $$\Delta G_{AB}=\int_{\lambda =0}^{\lambda =1}\frac{dG}{d\lambda }\;d\lambda =\int_{\lambda =0}^{\lambda =1}{\left\langle \frac{\partial V_{\lambda }}{\partial \lambda }\right\rangle }_{\lambda }\;d\lambda$$

When using FEP, instead, free energy differences are computed as

$$\Delta G_{AB}=-\beta^{-1}\;\ln\langle \exp\left(-\beta \Delta V_{AB}\right)\rangle_{A}^{eq}$$ 6

In drug discovery, alchemical transformations are frequently used to estimate relative binding free energies between analogous compounds A and B: *ΔΔG*_*b*_ = *ΔG*_*b*_(*B*) – *ΔG*_*b*_(*A*). To this end, ligand A is progressively transformed into ligand B, both bound to the protein and in the bulk. Subsequently, through a thermodynamic cycle, it is possible to estimate *ΔΔG*_*b*_.^12^

The first successful relative binding free energy calculation was performed by McCammon and co-workers in 1984.^13^ Today, this approach is still commonly employed to rank compounds with similar chemical structures, representing the predominant method used by pharmaceutical companies in lead optimization.^14^

More sophisticated thermodynamic cycles can be used to estimate absolute binding free energies (*ΔG*_*b*_). These alchemical approaches involve the transformation of the ligand into a fictitious noninteracting particle, effectively decoupling it from the protein and the bulk. Initially introduced by Jorgensen in 1988,^15^ this approach was refined and formalized by Gilson in 1997 ^16^ and is now commonly referred to as the double decoupling method.

Despite their robust theoretical foundations, alchemical methods present certain practical limitations. Achieving reliable convergence with FEP calculations can be challenging. In theory, FEP estimates can be obtained by sampling only equilibrium configurations starting from the reference state A to the target state B. For this reason, FEP is defined as a unidirectional equilibrium estimator. In practice, this would result in poor convergence, as for real systems there is usually a scarce overlap between the potential energy distribution of the initial and final states.^17^ Consequently, stratification involving intermediate states with 0 < λ < 1 values is often used.

To improve accuracy and efficiency in the estimation of *ΔG*_*AB*_, in 1976 Bennett demonstrated an optimal statistical method to combine information obtained from both forward and backward transformations, called the Bennett Acceptance Ratio (BAR) method.^18^ This method was also derived as a maximum-likelihood estimator for *ΔG*_*AB*_,^19^ by iteratively solving the following equation:

$$\langle {\left\{1+\exp\left[\beta \left(\Delta V-\Delta G_{AB}\right)\right]\right\}}^{-1}\rangle_{A}=\langle {\left\{1+\exp\left[-\beta \left(\Delta V-\Delta G_{AB}\right)\right]\right\}}^{-1}\rangle_{B}$$ 7

As forward and backward equilibrium transformations are combined, BAR is classified a bidirectional equilibrium estimator. Several alchemical protocols now rely on combining forward and backward transformations to improve results.^20,21^

Initially, alchemical transformations were primarily applied to small ligands, as large perturbations strongly hindered the convergence of FEP calculations. However, alchemical methods have since been extended to compute binding free energies of large and flexible ligands of pharmaceutical interest, such as the approved anticancer drug Gleevec with two kinase receptors: Abl tyrosine and c-Src.^22^

Approaches based on TI have also been used to estimate solvation free energies. Solubility is an important factor in drug development, as poor water solubility can significantly limit the bioavailability and absorption of a drug following oral administration.^23^ In our group, TI was used to evaluate the solubility advantage of amorphous ketoprofen relative to its crystalline form.^24^ In this work, the free energy required to transfer a ketoprofen molecule from its condensed phase (either amorphous or crystalline) into solution was evaluated with an ad-hoc approach that combines harmonic dynamics with MD simulations. Specifically, absolute free energies were computed as the sum of a reference free energy (comprising an analytically calculated ideal free energy term and harmonic contributions) and anharmonic corrections obtained via TI. Two thermodynamic cycles based on TI were used: the first connected the harmonic Hamiltonian to the anharmonic one at low temperature, while the second extended the results to 300 K for both amorphous and crystalline ketoprofen. The resulting free energies showed agreement with the experimental values, demonstrating the solubility advantage of the amorphous form.

Since their application in the lead optimization of anti-HIV-1 agents by Jorgensen in 2005,^25^ alchemical methods have remained the most common choice for computing binding free energies in the pharmaceutical industry. Nevertheless, the main advantage of alchemical transformations is also related to their limitation: because they sample the variation of the system through intermediates that have no physical relevance, these methods do not provide mechanistic or kinetic insight into the process under investigation.

## Path-Based Methods

Since the late 1970s, several research groups have focused on developing algorithms for free energy calculations based on physical pathways. This task is particularly challenging because many processes of interest in biochemistry and biophysics, including protein–ligand binding, are rare events. These rare processes are characterized by a very low probability of crossing high free energy barriers connecting narrow low energy regions in phase space. Consequently, observing these processes with standard MD simulations would require prohibitively long time scales that are computationally infeasible for practical use.

To overcome this limitation, methods that accelerate rare events and enhance conformational sampling have been developed. Over the years, several path-based enhanced sampling methods have been proposed, with notable examples including Umbrella Sampling, MetaDynamics, and Steered Molecular Dynamics.

### Umbrella Sampling

To mitigate the inefficiencies of standard MD, Torrie and Valleau introduced the Umbrella Sampling (US) method in 1977.^26^ In contrast to standard MD where the regular potential *V*(**q**) results in a Boltzmann-weighted sampling, US introduces a biasing potential in the form of a harmonic restraint centered at a specific value of the collective variable, ξ_0_:

8 $$\mathrm{V̂}\left(q\right)=V\left(q\right)+\frac{1}{2}k{\left(\xi \left(q\right)-\xi_{0}\right)}^{2}$$

When using a stratification strategy, multiple simulations are performed with the harmonic restraint centered at different CV values (ξ_0_). This bias enhances sampling in the selected regions of the configuration space, allowing the system to overcome free energy barriers. As a result, a non-Boltzmann sampling is obtained. To recover the unbiased Boltzmann distributions and determine the PMF, various postprocessing methods have been developed, with the Weighted Histogram Analysis Method (WHAM) being among the most widely used.^27,28^

Early applications of US include the calculation of the PMF of an ion pair in a polar fluid by Patey and Valleau.^29^ In the 1980s and 1990s, Karplus applied US to evaluate free energy differences in biological systems,^30^ while Jorgensen used it to study the conformational equilibria of *n*-Butane in dilute solution.^31^

In recent years, US has been used in various applications, including ligand-binding affinity prediction and reaction mechanism studies.^32,33^ In our group, we employed US with PCVs to explore the binding of a transition state analogue (DADMe-immucillin-H) to the purine nucleoside phosphorylase (PNP) enzyme.^34^ Initial binding pathways were identified by combining standard MD binding simulations with a machine learning (ML) algorithm. The binding free energy was then reconstructed using a nonlinear regression algorithm. Specifically, following the method of Maragliano et al.,^35^ we applied the Regularized Least Squares algorithm with a Gaussian kernel, using a Tikhonov regularization coefficient for numerical stability. This strategy produced a smooth free energy profile that accurately fit the data.

However, US strategies are complex and require the determination of two critical parameters: the force constants for harmonic restraints and the windows in the CV space for simulation. Due to these technical complexities, US-based methods are not widely adopted in drug discovery pipelines.

### MetaDynamics

MetaDynamics (MetaD) and MetaD-based approaches are among the most popular enhanced sampling methods for accelerating rare events. Introduced by Laio and Parrinello in 2002, MetaD allows systems to escape local free energy minima and explore the broader free energy landscape of rare events.^36^ This is achieved by applying a history-dependent bias potential to the dynamics of the system along one or more CVs. At regular intervals, small Gaussian potentials with width σ_*i*_ and height *W* are added at the current CV values, preventing the system from revisiting previously explored regions in the configuration space. Over time, this bias fills the free energy basins, leading to a diffusive behavior of the system along the CVs.^37^

The history-dependent bias at time *t* is given by:

$$V_{G}\left(\xi ,t\right)=\int_{t}^{0}\omega \;\exp\left(-\overset{d}{\underset{i=1}{\sum }}\frac{{\left(\xi_{i}\left(t\right)-\xi_{i}\left(\tau \right)\right)}^{2}}{2{\sigma_{i}}^{2}}\right)\;d\tau$$ 9

where *d* is the dimension of the CV space and ω is an energy rate calculated as the ratio of the Gaussian height *W* and the deposition stride τ_*G*_. Once MetaD reaches convergence, the bias potential becomes an unbiased estimator of the PMF, according to the following relation:

10 $$V_{G}\left(\xi ,t\to \infty \right)=-G\left(\xi \right)+C$$

where *C* is an arbitrary constant.^38^

The initial implementation of MetaD struggled with convergence issues, leading to the development of Well-Tempered MetaD (WT-MetaD)^39,40^ and further refined variations. WT-MetaD was first applied to protein–ligand binding studies by Gervasio et al.^41^ and remains a valuable tool in drug design to thoroughly investigate binding mechanisms and compute PMFs. A comprehensive review of MetaD-based algorithms in drug discovery, particularly in the analysis of ligand-protein association and dissociation mechanisms, can be found in ref 42.

Our research group has widely employed MetaD and MetaD-related methods in the context of rational drug design.^41,43–45^ In particular, in 2009 we used WT-MetaD to refine docking poses, accounting for molecular flexibility and exploring binding mechanisms.^44^ This successful protocol not only improves the predictive accuracy of docking but also reveals the plausible unbinding mechanisms of compounds of interest. Nevertheless, the majority of MetaD applications have been retrospective, while predictive/prospective studies are rather limited. In an effort to improve and automate MetaD approaches for computing binding free energies, in our group we developed a protocol that integrates enhanced sampling, ML, and specialized algorithms to minimize computation time and the number of free parameters in free energy calculations.^1^ This protocol was applied in a prospective study of ligands binding to a protein kinase of pharmacological interest, glycogen synthase kinase 3β, achieving strong agreement with experimental values. This work represents the state-of-the-art of MetaD-related methods in our group.

### Steered Molecular Dynamics

SMD is an enhanced sampling method developed by the group of Schulten at the end of the 1990s.^46,47^ Similarly to US, in SMD a harmonic biasing potential is added to the standard potential of the systems. However, in SMD, the center of the external potential is not fixed, but evolves over time along the CV (ξ), driving the system out of equilibrium:

11 $$V_{\lambda }\left(\xi ,t\right)=\frac{1}{2}k{\left(\xi -\xi_{0}\left(t\right)\right)}^{2}$$

This time dependence makes SMD a nonequilibrium method.

SMD was developed to investigate biological rare events, including protein folding^48^ and protein–ligand binding.^49^ In the latter case, the unbinding event is enhanced by attaching a virtual spring with force constant *k* to the ligand and pulling it along a CV (often the distance between the ligand and the pocket) with a finite constant velocity *v*. As SMD is a nonequilibrium method, results obtained will highly depend on the parameters *k* and *v*, requiring their fine-tuning.

From SMD simulation, the PMFs and *ΔG*_*AB*_ can be estimated using the Jarzynski equality, which relates the nonequilibrium work (*W*_*J*_) to the equilibrium free energy difference:^50^

$$\exp\left(-\beta \Delta F_{AB}\right)=\langle \exp\left(-\beta W_{J}^{f}\right)\rangle_{A\to B}$$ 12

The angular brackets specify an exponential average over trajectories of the system evolving from state A to B with the same protocol. Therefore, the Jarzynski equality is a unidirectional estimator and can be seen as the nonequilibrium version of FEP (eq 6).

The first application of SMD as a drug discovery tool can be attributed to our group, with the work by Colizzi et al.^51^ in 2010. In this study of antimalarial drug discovery, SMD was used to investigate molecular interactions and analyze the binding characteristics of flavonoids. By examining the force profiles derived from ligand unbinding simulations, SMD successfully differentiated between active and inactive enzyme inhibitors. Furthermore, PMFs were defined using the Jarzynski second cumulant expansion,^50^ enabling a comprehensive *in silico* characterization of ligand analogues. This work laid the foundation for new structure-based drug design strategies leveraging SMD.

Building on this study, our group presented an innovative postprocessing approach for analyzing nonequilibrium SMD trajectories.^52^ This method aimed to identify key structural features relevant to the binding process without relying on the Jarzynski estimator for PMF reconstructions, which often requires substantial computational resources. Instead, we showed that valuable structural insights could be obtained from small sets of pulling trajectories. This was possible because SMD work profiles retain crucial information about structural events, even in an out-of-equilibrium context.

Using the fine-tuned SMD parameters determined in this study, we subsequently applied SMD to investigate the unbinding mechanisms of nine kinase inhibitors.^53^ Ligands were evaluated in terms of the decay of rupture force profiles over simulation time, the unbinding times, and the PMFs. SMD was able to successfully distinguish active from inactive inhibitors within congeneric series; however, it was less effective at ranking ligands with similar potencies across different chemical classes. Nevertheless, SMD provided key atomistic insights into the binding mechanisms of kinase inhibitors, which are significantly challenging due to their flexible and solvent-exposed active sites.

Finally, the most recent application of SMD simulations in our group is presented in ref 2. Building on the concepts introduced in ref 1, this study explores the integration of SMD nonequilibrium approaches with PCVs to reconstruct the PMF and calculate absolute binding free energies. Considering the convergence issues associated with the Jarzynski equality, in this work free energy differences were instead obtained using the Crooks Fluctuation Theorem (CFT),^54^ which combines data from forward and backward transformations. Like the BAR method, CFT is a bidirectional estimator, offering improved accuracy and convergence compared to the unidirectional Jarzynski equality. While BAR focuses on potential energy differences and CFT on Jarzynski nonequilibrium work, the two are mathematically equivalent.^55^ This similarity extends to the FEP and Jarzynski estimators, which can be viewed as special cases of the Crooks Fluctuation Theorem.^19,56^

Like the BAR method, CFT can be expressed through a maximum likelihood approach:^19^

$$\langle {\left\{1+\exp\left[\beta \left(W_{J}^{f}-\Delta G_{AB}\right)\right]\right\}}^{-1}\rangle_{A\to B}=\langle {\left\{1+\exp\left[-\beta \left(W_{J}^{b}-\Delta G_{AB}\right)\right]\right\}}^{-1}\rangle_{B\to A}$$ 13

The newly proposed semiautomated workflow yields reliable results for absolute binding free energy, while also providing a detailed picture of dissociation mechanisms at the molecular level. The great advantage of this strategy lies in the straightforward parallelization of SMD replicas, in contrast to methods based on intrinsically serial algorithms, such as MetaD.

Additional studies from our group on the role of dissipation revealed practical strategies to mitigate work dissipation and improve the convergence of free energy estimates.^57^ This enabled the application of our nonequilibrium approach to challenging and intricate systems, including the pharmaceutically relevant Abl-Gleevec complex and RNA-ligands complexes.^10^

## Challenges and Perspective

In the current Account, we summarized MD-based approaches used in drug discovery to calculate binding free energies. As discussed, free energy calculations relying on MD simulations primarily fall into two main categories: alchemical transformations and path-based (geometrical) methods. These approaches differ in their underlying principles: alchemical transformations rely on nonphysical coupling parameters, whereas path-based methods use physically meaningful collective variables. In ref 20, the authors present two formal routes for computing binding free energies: one based on alchemical transformations (double decoupling) and the other using physical collective variables. Although distinct, both approaches share a similar framework, relying on restraints to facilitate sampling convergence, ultimately yielding equivalent results with a comparable computational cost.^20^ The choice between the two typically depends on the specific problem studied.

Alchemical transformations are particularly well suited for evaluating a large number of complexes, such as in the hit-to-lead step to rank potential compounds based on binding affinity. Moreover, alchemical transformations are beneficial when no prior knowledge of suitable CVs is available. Nevertheless, alchemical transformations are often limited to small and moderately charged ligands, due to convergence difficulties associated with large perturbations and substantial solvation free energies. Additionally, a key limitation is that alchemical computations cannot provide mechanistic details about the process.

In contrast, path-based methods can capture the mechanistic details and give insight into the kinetics of the transformation. However, the selection of appropriate CVs is often nontrivial, and nonoptimal choices may lead to poor sampling and convergence issues. Committor analysis can therefore be used to assess how well a CV captures the reaction coordinate and the key features of drug binding and unbinding. Nonetheless, methods based on physical pathways remain the most suitable approach for studying the binding of large ligands and, in particular, protein–protein binding. Due to their extensive computational cost, path-based methods find their application in drug discovery during the lead optimization stage of the most promising compounds.

A promising alternative to pure alchemical transformation and physical route methods may be represented by the Alchemical Transfer Method (ATM), recently developed by Gallicchio and collaborators.^58^ ATM is based on a coordinate displacement perturbation of the ligand between the receptor binding site and the explicit solvent bulk. ATM occupies an intermediate position in the landscape of free energy methods, combining the nonphysical transformations of alchemical approaches (such as double decoupling) with structural features that resemble physical routes used in path-based methods.

Another interesting approach is Hamiltonian Replica Exchange MD (H-REMD), in which multiple replicas of the system are simulated in parallel using modified Hamiltonians (usually, by scaling nonbonded parameters describing interparticle interactions). Periodically, exchanges between replicas are attempted to improve the sampling of intermediate states and convergence of both alchemical and geometrical route methods.^59–61^

Overall, MD-based approaches and free energy computations have the potential to become effective tools for rational drug design. Nevertheless, despite significant advances, some limitations persist. The primary challenges are related to (i) the difficulty of extensively sampling the protein–ligand binding rare event, (ii) the inaccuracies of current force fields (FFs), and (iii) convergence issues.

When high-probability regions of the configuration space are separated by significant free energy barriers, as in the case of protein–ligand binding, standard MD techniques often struggle to sample all relevant states within feasible time scales. This phenomenon, known as quasi-nonergodicity, necessitates the use of enhanced sampling methods, which aim to overcome free energy barriers and enable a more comprehensive sampling of rare but critical events. Today, a wide range of enhanced sampling methods is available, each with its own inherent advantages and limitations. Many of these methods rely on carefully chosen CVs to improve the sampling in regions of interest. In this context, ongoing advances in the design of more advanced CVs, including those powered by ML, specifically tailored for drug discovery, are helping to overcome these sampling challenges.^62^

Another key aspect to consider is that computer simulations generate microscopic structural ensembles with statistical weights governed by the theoretical model applied. In protein–ligand simulations, this model is typically a FF: a set of parameters and mathematical functions that describe intra- and intermolecular interactions. Consequently, the accuracy of simulation results is intrinsically tied to the quality of the FF used. To address current limitations, ML-based FFs have emerged as a promising avenue for improving the representation of potential energy surfaces. By combining accuracy and computational efficiency, ML-based FFs have the potential to broaden and enhance the applicability of MD-based methods in drug discovery. Notably, our group has recently developed a novel Neural Network Potential (OBIWAN), which may represent a significant step forward in this direction.^63^

Finally, convergence issues, although often underestimated, can significantly impact the reliability of computational results. These issues may arise in various contexts. For example, poor convergence is common in nonequilibrium simulations using the Jarzynski equality, where the estimate is dominated by rare trajectories with low work values. Similarly, convergence issues in sampling, as often encountered in MetaD simulations, can lead to unstable free energy estimates. Ensuring convergence is therefore crucial to obtain robust and reproducible results. This calls for the definition of general guidelines to assess convergence of MD simulations. Verifying the stability of results over the simulation time is a straightforward and necessary, although not sufficient, metric of convergence. However, defining precise guidelines is challenging, as different methods rely on distinct estimators and may require different convergence criteria. In the case of free energy estimators, rigorous statistical analyses are essential for evaluating convergence. For example, in ref 2, we presented a comprehensive study on the convergence of free energy calculations, highlighting the importance of systematic convergence assessments to ensure accurate and reliable computational outcomes.

All of the advancements discussed will foster the broader adoption of MD methodologies in drug discovery. While static molecular docking approaches are effective in the earliest stages of drug discovery for hit identification, binding free energy methods provide a more rigorous refinement of results and become valuable in the hit-to-lead and lead optimization phases. In particular, MD-based dynamical approaches can be systematically integrated into routine docking workflows (e.g., through dynamic docking),^64^ improving static or semiflexible algorithms in the key steps of posing and scoring. This shift will enable the computational community to extensively adopt more accurate tools for predicting the structure and energetics of drug-target complexes, thereby accelerating the identification, optimization, and development of novel bioactive compounds and ultimately transforming the drug discovery process.
